# The cognitive dysfunction related to Alzheimer disease or cerebral small vessel disease: What's the differences

**DOI:** 10.1097/MD.0000000000026967

**Published:** 2021-08-27

**Authors:** Zhenhong Liang, Lijuan Wu, Shumei Gong, Xiaohong Liu

**Affiliations:** a School of Medicine, Taizhou University, Zhejiang Province, Taizhou 318000, China; b School of Nursing, The second Military Medical Universtiy, Shanghaihai 2000433, China.

**Keywords:** Alzheimer disease, cerebral small vessel disease, cognitive dysfunction, risk

## Abstract

Alzheimer disease (AD) and sporadic cerebral small vessel disease (CSVD) are common cognitive disorders. Both AD and CSVD have mental symptoms including chronic progressive cognitive impairment, dysfunction, and behavioral abnormalities. However, the differences on the cognitive dysfunction of AD and CSVD remain unclear. It is necessary to elucidate the cognitive dysfunction differences of AD and CSVD, and to identify the potential risk factors.

AD or sporadic CSVD patients treated in our hospital from December 1, 2018 to May 31, 2019 were included. And we selected healthy participants as controls. The mini-mental state examination and Montreal Cognitive Assessment Scale were used for neuropsychological assessment, and related medical information were collected and compared.

A total of 190 patients were included. The total mini-mental state examination scores in AD, CSVD group were significantly less than that of control group, there were significant differences in the domains of directional ability, attention and computing ability, delayed recall, and visual perception (all *P* < .05); the total Montreal Cognitive Assessment Scale scores in AD, CSVD group were significantly less than that of control group. There were significant differences in the domains of visual space and execution, immediate remember, attention and computing ability, language, delayed recall, and directional ability (all *P* < .05); diabetes was a risk factor both for AD (hazard ratio = 1.63, 95% confidence interval: 1.35–1.97) and CSVD (hazard ratio = 1.15, 95% confidence interval: 1.08–1.27).

The cognitive dysfunctions of AD are difference to that of CSVD patients, and diabetes is the risk factor both for AD and CSVD, future studies are needed to further identify the prevention and treatment of AD and CSVD.

## 1 Introduction

Alzheimer disease (AD) is a common neurological degenerative disease in the elderly, and it has been reported that AD is the most common cause of cognitive impairment, and its incidence gradually increases with the increase of age.^[1,2]^ The prevalence of AD can double with every 6.1 years of age increase.^[3]^ The etiology of AD is unclear so far, its onset may be related to the abnormal deposition of Aβprotein in the brain, which has toxic effects on neurons, and it can cause the loss of various neurotransmitters such as acetylcholine in the brain, and eventually lead to cognitive dysfunction.^[4]^ There are many known risk factors for AD patients, the most important of which are age, family history AD, and apolipoprotein E genotype.^[5]^ In addition, smoking, drinking, education, hypertension, diabetes et al may also affect the occurrence and development of AD.^[6]^ The pathology of AD patients is diffuse brain atrophy, especially in the parietal and pre-frontal brain, and this pathological change increases with the degree of lesions.^[7]^ Microscopically, it can been seen that senile plaque formation, neuron fiber tangles with granular vacuole degeneration and extensive neuronal loss.^[8]^ In the early stages of the disease, patients with AD mainly manifest as memory impairment, and near memory is mainly impaired. Although research and understanding of AD have continued to deepen in recent years, no cure has been found that can effectively curb AD progress.^[9]^ The patient's condition can progress significantly and its course usually ranges from 8 to 10 years.^[10]^ It has been reported that AD patients eventually die from complications such as malnutrition, secondary infections, and deep vein thrombosis.^[11]^ Therefore, the early detection of cognitive dysfunction for the early treatment of AD is critically important to the prognosis of AD patients.

Cerebral small vessel disease (CSVD) refers to the pathological manifestations caused by the lesions of intracranial small vessels.^[12]^ It can easily cause various impairments such as cognitive dysfunction, dementia, depression, micturition, and abnormal gait. The pathologically images are presented as the lacunar infarction, subcortical white matter lesions, cerebral hemorrhage, and microinfarction.^[13]^ It has been reported^[14]^ that the most common risk factors for arteriosclerotic CSVD are age, diabetes, and hypertension, of which hypertension is the most relevant. The status of cognitive function related to CSVD includes the severity of cognitive dysfunction at various stages from normal cognitive function to dementia, which is related to the type, location, and extension of lesion.^[15]^ In clinical practice, the scales currently used in the evaluation of cognitive function are mainly mini-mental state examination (MMSE) and Montreal Cognitive Assessment Scale (MoCA). There is currently no unified neuropsychological scale suitable for sporadic CSVD cognitive impairment. Therefore, to further elaborate the characteristics of cognitive dysfunction caused by sporadic CSVD, and to establish a highly sensitive and operable predictive scale is one of directions of future researches on CSVD.

Cognitive dysfunction not only causes the patient's occupational and social abilities to decline or even be lost, it also causes heavy physical and mental stress to caregivers, and it also brings huge economic pressure to patients’ families and society.^[16,17]^ According to statistical reports,^[18]^ in China the cost of cognitive impairment can increase from US$ 47.2 billion in 2010 each year to US $69 billion in 2020. As a common disease of the elderly, AD and sporadic CSVD is manifested by chronic, progressive cognitive decline. Both AD and CSVD have neurological symptoms including chronic progressive cognitive impairment, dysfunction, and behavioral abnormalities.^[19,20]^ And the patient's condition can progress significantly and its course usually ranges from 8 to 10 years.^[21]^ It is necessary to take early measures to combat the progress of AD and sporadic CSVD. Therefore, we aimed to conduct this study to compare the characteristics of cognitive impairment of AD and sporadic CSVD, thereby providing evidences for the differential diagnosis and early treatments of AD and sporadic CSVD.

## 2 Methods

### 2.1 Ethical consideration

Our study had been approved by the ethics committee of our hospital (approval number: 20180920), and the written informed consents were obtained from all included participants.

### 2.2 Participants

AD or sporadic CSVD patients who were treated in the department of neurology of our hospital from December 1, 2018 to May 31, 2019 were included. The inclusion criteria were as following: the diagnoses of AD or sporadic CSVD complied with the clinical guidelines^[22,23]^ of AD or sporadic CSVD; the degree of cognitive impairment was mild to moderate (13 < MMSE < 24); the patients signed the informed consents and were willing to inclusion for future follow-up. At the same period, we included those who came to our hospital for normal neuropsychology and head magnetic resonance imaging (MRI) examination as control group, the MMSEs of control groups were all >27. The exclusion criteria were as following: patients with family history of Alzheimer, a large area of cerebral infarction or cerebral hemorrhage or brain trauma; patient with a history of anxiolytic or antidepressant use; patients with diseases that can cause cognitive decline such as thyroid dysfunction, vitamin deficiency, Parkinson disease, multiple sclerosis.

### 2.3 Data collections

We firstly patients with AD or sporadic CSVD patients by searching the keywords of patients’ diagnosis in the medical record system, then 2 authors screened for the retrieved patients according to the inclusion and exclude standards. At the time of enrollment, we collected the related medical information from included participants, including the patient age, gender, the history of smoking, drinking. Furthermore, we detected and collected the current healthy status of participants including hypertension, diabetes, hyperlipidemia, homocysteine.

### 2.4 Neuropsychological assessment

The MMSE and MoCA were used for neuropsychological assessment in this present study. The MMSE scale^[24]^ has a total of 30 scores, including 10 scores of directional ability (temporal and spatial directional ability), 3 scores of immediate memory, 5 scores of attention and computing ability, 3 scores of delayed recall, and 8 scores for language (denomination, retell, read, comprehension, and writing), 1 score for visual perception. The MoCA scale^[25]^ has a total of 30 scores, including language skills (denomination, repetition, fluency, summary), attention and computing ability (orderly read, reversely read, computing ability) and directional ability with 6 scores for each domain, 5 scores for delayed recall, 5 scores for visual space and execution (cube copy test, line connection test, picture clock test). Two authors evaluated the patients independently, and any conflicts were solved with further discussions.

All patients underwent neurological screening and head MRI after admission. We use the 1.5T superconducting MRI scanner and special head coil produced by General Motors for inspection. ① Image acquisition: The sequence was T1W1 and T2W1. T1W1: Repeat: 9. 6 ms, echo: 4. 5 ms, interval: 5 mm, layer thickness: 1 mm, flip angle 10°, field of view 210, matrix 195 × 175. T2W1: Repeat: 3070 ms, echo: 75 ms, interval: 1 mm, layer thickness: 5 mm, flip angle 90°, field of view 240, matrix 290 × 170. We selected the horizontal axis and the sagittal for scanning, the horizontal positioning line is parallel to the line of the lower edge of the front and back of the brain, the sagittal position was the positioning line parallel to the brain, and the light form was the positioning line parallel to the hippocampus axis; ② Building a test network: we set according to the proportion of the patient's head MRI film, then place the test grid on the MRI diagnostic film for accurate counting, calculated the hippocampal volume according to the Cavalieri cause of the measured object volume formula, and used the PACS measurement tool to measure the hippocampal height and the angular width, sylvian fissure width, frontal angle index, and caudate nucleus index; ③ Severity of leukoaraiosis: 0 points: no lesions on both sides, 1 point: 1 to 2 lesions, 2 points: 3 to 5 lesions, 3 points: 5 or more lesions, 4 points: fusion lesions; ④ Auxiliary examination: AD lesions existed not only in gray matter, but also in white matter. DTI was used to analyze the increase in the dispersion of white matter water molecules in the frontal lobe, temporal lobe, and parietal lobe.

### 2.5 Statistical analysis

All statistical analyses were performed with SPSS 23.0 software (IBM, USA). The measurement data were presented with mean ± standard deviation ($\bar{\text{x}}\pm \text{SD}$). Comparisons between consecutive variables were performed using an independent sample *t* test; The categorical variables were expressed as ratio (%), and the comparisons between categorical variables were performed using a chi-square test. Furthermore, to assess the potential risks of clinical endpoints, the Cox proportional hazard regression model was used to calculate the hazard ratio and the 95% confidence interval. *P* < .05 was considered as the difference being statistically significant.

## 3 Results

### 3.1 The characteristics of included participants

A total of 190 patients were included in this present study, with 62 AD patients, 63 CSVD patients, and 65 healthy controls respectively. As presented in Table 1, there were significant differences in the number of smoking, hypertension, and diabetes (all *P* < .05), no significant differences were found in the age, gender, and number of hyperlipidemia and high-Hcy (all *P* > .05).

**Table 1:** The characteristics of included participants.

Items	AD group (n = 62)	CSVD group (n = 63)	Control group (n = 65)	t/χ^2^	*P*
Age (yr)	73.6 ± 5.05	73.2 ± 5.18	72.9 ± 5.47	19.085	.191
Female/male	30/32	30/33	32/33	1.327	.464
Smoking (%)	27 (43.55%)	27 (42.86%)	10 (15.38%)	1.058	.045
Drinking (%)	38 (61.29%)	40 (63.49%)	29 (44.62%)	1.182	.088
Hypertension (%)	26 (41.94%)	40 (63.49%)	15 (23.08%)	0.996	.029
Diabetes (%)	14 (22.58%)	29 (46.03%)	8 (12.31%)	1.409	.034
Hyperlipidemia (%)	12 (19.35%)	16 (25.39%)	10 (15.38%)	1.157	.091
High-Hcy (%)	11 (17.74%)	13 (20.63%)	10 (15.38%)	0.951	.135

AD = Alzheimer disease, CSVD = cerebral small vessel disease.

### 3.2 The MMSE assessment

As presented in Table 2, the total MMSE scores in AD, CSVD group were significantly less than that of control group. There were significant differences in the domains of directional ability, attention and computing ability, delayed recall and visual perception (all *P* < .05), no significant differences were found in the domains of immediate memory and language (all *P* > .05).

**Table 2:** The MMSE scores distribution among 3 groups.

Items	AD group (n = 62)	CSVD group (n = 63)	Control group (n = 65)	t	*P*
Directional force	5.03 ± 1.74^∗^	6.09 ± 1.14^∗^^,^^#^	8.25 ± 1.09	6.985	.009
Immediate memory	2.75 ± 0.85	2.65 ± 0.79	2.89 ± 0.99	3.011	.184
Attention and computing ability	2.78 ± 0.92^∗^	2.24 ± 0.90^∗^^,^^#^	4.17 ± 1.02	8.095	.036
Delayed recall	0.54 ± 0.19^∗^	0.56 ± 0.11^∗^	2.06 ± 0.46	4.423	.013
Language					
Denomination	2.00 ± 0.00	2.00 ± 0.00	2.00 ± 0.00	1.000	1.000
Retell	0.76 ± 0.19	0.50 ± 0.15^∗^^,^^#^	0.77 ± 0.18	1.498	.985
Read	0.82 ± 0.21	0.79 ± 0.28^#^	0.81 ± 0.15	1.845	.092
Comprehension	2.48 ± 0.53	2.13 ± 0.89^∗^^,^^#^	2.50 ± 0.55	1.004	.145
Writing	0.46 ± 0.28	0.39 ± 0.22^∗^	0.55 ± 0.26	0.593	.068
Visual perception	0.62 ± 0.20^∗^	0.76 ± 0.15^∗^	1.00 ± 0.00	1.343	.012
Total scores	18.69 ± 5.14^∗^	17.83 ± 4.96^∗^	26.34 ± 6.97	19.38	.007

AD = Alzheimer disease, CSVD = cerebral small vessel disease, MMSE = mini-mental state examination.

∗ *P* < .05 when compared with control group.

# *P* < .05 when compared with AD group.

### 3.3 The MoCA assessment

As presented in Table 3, the total MoCA scores in AD, CSVD group were significantly less than that of control group. There were significant differences in the domains of visual space and execution, immediate remember, attention and computing ability, language, delayed recall and directional ability (all *P* < .05), no significant difference was found in the domain of denomination (*P* = .141).

**Table 3:** The MoCA scores distribution among 3 groups.

Items	AD group (n = 62)	CSVD group (n = 63)	Control group (n = 65)	t	*P*
Visual space and execution	2.43 ± 1.01^∗^	1.29 ± 0.25^∗^^,^^#^	3.55 ± 1.13	1.995	.004
Cube copy	0.57 ± 0.12^∗^	0.23 ± 0.19^∗^^,^^#^	0.95 ± 0.24	1.459	.015
Line connection test	0.09 ± 0.02^∗^	0.00 ± 0.00^∗^	0.53 ± 0.21	1.090	.007
Picture clock test	1.83 ± 0.75^∗^	0.97 ± 0.21^∗^^,^^#^	2.44 ± 0.79	1.453	.042
Denomination	2.32 ± 0.95^∗^	2.29 ± 0.94^∗^	2.51 ± 0.81	1.118	.141
Immediately remember (phase I)	2.08 ± 0.77^∗^	2.01 ± 0.90^∗^^,^^#^	3.64 ± 1.07	2.085	.035
Immediately remember (phase II)	3.45 ± 0.85^∗^	3.17 ± 1.04^∗^^,^^#^	4.81 ± 0.98	3.552	.028
Attention and computing ability	3.96 ± 0.95^∗^	3.19 ± 1.35^∗^^,^^#^	4.35 ± 0.72	1.989	.015
Orderly read	0.87 ± 0.14^∗^	0.73 ± 0.18^∗^	1.00 ± 0.00	1.085	.041
Reversely read	0.79 ± 0.21	0.48 ± 0.11^∗^^,^^#^	0.90 ± 0.13	1.459	.022
Attention	0.53 ± 0.15^∗^	0.24 ± 0.08^∗^^,^^#^	0.89 ± 0.35	0.980	.028
Computing ability	2.04 ± 0.97^∗^	1.75 ± 0.12^∗^	2.99 ± 0.84	1.442	.044
Language	1.38 ± 0.17^∗^	0.85 ± 0.28^∗^^,^^#^	2.45 ± 0.92	1.408	.039
Repeat	1.02 ± 0.09^∗^	0.79 ± 0.21^∗^^,^^#^	2.49 ± 0.85	0.986	.009
Fluency	0.28 ± 0.14^∗^	0.15 ± 0.09^∗^^,^^#^	0.88 ± 0.30	1.152	.028
Summary	0.79 ± 0.11^∗^	0.28 ± 0.09^∗^^,^^#^	1.07 ± 0.37	0.827	.017
Delayed recall					
Free recall	0.29 ± 0.07^∗^	0.22 ± 0.05^∗^	2.99 ± 0.85	1.104	.009
Directional ability	3.58 ± 1.02^∗^	3.75 ± 0.95^∗^^,^^#^	5.22 ± 1.17	2.251	.015
Total scores	14.62 ± 4.97^∗^	11.69 ± 3.19^∗^^,^^#^	24.59 ± 6.51	10.352	.011

AD = Alzheimer disease, CSVD = cerebral small vessel disease, MoCA = Montreal Cognitive Assessment Scale.

∗ *P* < .05 when compared with control group.

# *P* < .05 when compared with AD group.

### 3.4 Cox risk regression analysis on the risk factors of AD

As presented in Table 4, diabetes was the risk factor for the development of AD in patients with AD (hazard ratio = 1.63, 95% confidence interval: 1.35–1.97). Age, gender, hypertension, drinking, smoking, hyperlipidemia and high-Hcy were not related to the development of AD (all *P* > .05).

**Table 4:** Cox risk regression analysis on the risk factors of AD.

Variables	Univariate regression		Multivariate regression	
	HR (95%CI)	*P*	HR (95%CI)	*P*
Age	1.04 (1.00–1.08)	.184	–	–
Female	1.74 (0.96–2.45)	.130	–	–
Hypertension (%)	1.19 (1.01–1.38)	.047	1.10 (0.94–1.37)	.099
Drinking (%)	1.01 (0.93–1.09)	.098	–	–
Smoking (%)	1.09 (0.87–1.05)	.079	–	–
Diabetes (%)	1.48 (1.04–1.98)	.042	1.63 (1.35–1.97)	.015
Hyperlipidemia (%)	1.90 (0.82–1.05)	.195	–	–
High-Hcy (%)	1.14 (0.66–3.58)	.507	–	–

AD = Alzheimer disease, CI = confidence interval, HR = hazard ratio.

### 3.5 Cox risk regression analysis on the risk factors of CSVD

As presented in Table 5, diabetes was the risk factor for the development of CSVD in patients with CSVD (hazard ratio = 1.15, 95% confidence interval: 1.08–1.27). The age, gender, hypertension, drinking, smoking, hyperlipidemia and high-Hcy were not related to the development of AD (all *P* > .05).

**Table 5:** Cox risk regression analysis on the risk factors of CSVD.

	Univariate regression		Multivariate regression	
Variables	HR (95%CI)	*P*	HR (95%CI)	*P*
Age (yr)	1.05 (1.01–1.08)	.149	–	–
Female	1.75 (0.95–2.45)	.197	–	–
Hypertension (%)	1.17 (1.09–1.32)	.039	1.35 (0.90–1.65)	.204
Drinking (%)	1.07 (0.96–1.14)	.253	–	–
Smoking (%)	1.15 (0.82–1.27)	.094	–	–
Diabetes (%)	1.02 (1.01–1.05)	.015	1.15 (1.08–1.27)	.043
Hyperlipidemia (%)	1.29 (0.76–1.76)	.368	–	–
High-Hcy (%)	1.24 (0.64–2.31)	.315	–	–

CI = confidence interval, CSVD = cerebral small vessel disease, HR = hazard ratio.

## 4 Discussion

AD is a neurological degenerative disease mainly manifested by acquired progressive cognitive impairment, sleep disturbance, mental and behavioral abnormalities.^[26]^ The cognitive domain involved in the early stage of the disease is mainly delayed recall. When the disease progresses to the middle and late stages, the delayed recall is completely lost and damage to other cognitive domains such as orientation, attention and calculation, and visual spatial perception.^[3,27]^ CSVD is a syndrome caused by intracranial small vessel injury, and its condition changes gradually.^[28]^ Both AD and CSVD need early interventions and treatments for further adverse progress. It can easily cause various impairments such as cognitive dysfunction, dementia, depression, micturition(?), and abnormal gait. The results of this present study have revealed that compared with AD, the CSVD has severe damages in the domains of immediate memory, attention and computing ability, language fluency, abstract thinking and executive ability, but the performance in directional ability for CSVD is better than AD, and no significant differences have been found in the domains of denomination, retell and free recall. Furthermore, we have identified that diabetes was the risk factor both for the development of AD and CSVD.

AD is a representative of cortical dementia, and the main pathological change is atrophy of the cerebral cortex.^[29]^ The most classic manifestation of AD in structural imaging is the change of the medial temporal lobe, especially the hippocampus and entorhinal cortex.^[30]^ When structural changes have not occurred in the early stage of AD, local blood flow or metabolic activity has changed.^[31]^ Functional imaging can improve the specificity of early diagnosis of AD.^[32]^ Therefore, some scholars have incorporated PET functional images into the AD diagnostic criteria. Studies^[33–35]^ have found that in patients with AD, the PET imaging of low-metabolized regions are mainly found in the posterior cingulate gyrus and the anterior lobes, while the lesions in patients with early-onset AD are distributed in the parietal, occipital, and frontal cortex and subcortical regions. It has been reported^[36]^ that Ap amyloid imaging can effectively distinguish AD from other dementia types. For pathologically confirmed AD patients, the accuracy of Pittsburgh compound imaging is as high as 69% of patients with subcortical vascular dementia.^[37]^ MRI findings between the subgroups. Since our study was a retrospective study design, some data regarding MRI were missing, the MRI findings between the subgroups is vital to detect the group differences, Therefore, imaging differences should be analyzed distinguish AD from CSVD in future studies.

AD and sporadic CSVD have different performances in different cognitive domains. The former is based on episodic memory impairment, while the latter is mainly manifested in attention loss, decreased information processing speed, and executive dysfunction.^[38]^ AD patients suffer from information storage and coding disorders due to hippocampal-medial leaf atrophy, so AD can be manifested as a cryptic onset of antegrade episodic memory disorder.^[39]^ AD patients often cannot recall what just happened, and even cues provided by other people cannot AD patients recall what have just happen, impaired episodic memory is also one of the most likely diagnostic criteria for AD.^[40]^ Compared with AD, the damage of the frontal-subcortical loop in patients with sporadic CSVD mainly affects the extraction and reproduction of information, keeps the memory function relatively, and the delayed recall score is often better than that of AD, and with clues CSVD patients’ recognition can be improved.^[41]^ Early AD patients have complex impaired attention, but the lack of attention maintenance and concentration will not appear until the later stages.^[42]^ Therefore, early AD patients can achieve higher results by performing a digital span test. Patients with CSVD also have impaired attention. AD's executive dysfunction is manifested by a lack of attention maintenance, a weakened ability to suppress interference, and a decline in reasoning ability.^[43]^ Although some studies^[44–46]^ have found that cognitive impairment caused by sporadic CSVD can have significant memory impairment, executive dysfunction is currently considered to be a characteristic of sporadic CSVD. And some studies^[47,48]^ have shown that the quality of life of patients with CSVD is related to impairment of executive function. The language impairment in AD patient is manifested in the early stages as difficulty in finding and organizing words and sentences, but there is no damage to words repetition and pronunciation.^[49]^ With the progress of the disease, reading and writing ability further decreased, and finally patients may make stereotyped speech.^[50]^ Nevertheless, the language disorder of the scattered CSVD is often manifested as less speech, but the understanding and expression ability are generally intact. Previous study^[51,52]^ has compared the language functions of patients with AD and CSVD, and it has been found that the AD group only has showed impairment of semantic fluency, while the CSVD group has showed impaired semantic fluency and speech fluency, which may be related to the reduction of executive function and processing speed.

Identifying the risk factors for AD and CSVD is important for its prevention and treatment. The pathogenesis of AD has not been fully elucidated. Environmental factors and genetic susceptibility play important roles in its pathogenesis, such as: age, diabetes, hypertension, hypercholesterolemia, smoking and drinking, and other unhealthy lifestyles.^[53]^ But among the risk factors for sporadic CSVD, age, hypertension, and diabetes are the most common risks factors.^[54]^ The results of this present study have identified diabetes as the risk factor both for AD and CSVD. Under the influence of vascular risk factors such as diabetes, the arteriosclerosis worsens, and pathological changes such as cellulosic necrosis and amyloidosis appear, thus eventually leading to brain parenchymal damage. Previous studies^[55,56]^ have identified that the age, hypertension, and diabetes are risk factors for cognitive decline caused by sporadic CSVD. Among them, age is an uncontrollable factor for us, while the blood pressure and blood glucose levels are controllable risk factors. Early detection and standardized treatment of hypertension and diabetes are particularly important to reduce the development of cognitive impairment of AD and sporadic CSVD patients.^[57]^

## 5 Conclusions

In conclusion, we have found that compared with AD, CSVD has severe impairments in the area of immediate memory, attention and calculation ability, language fluency, abstract thinking and execution ability, but the orientation ability of CSVD patient is better than that of AD patient. Besides, we have identified diabetes as a risk factor both for AD and CSVD development. However, limited by the sample size and the limited tools of neuropsychological assessment, more studies in the future are needed to further elucidate the cognitive dysfunctions in AD and CSVD patients.

## Author contributions

Z L designed research; Z L, L W conducted research; Z L, S G, X L analyzed data; Z L wrote the first draft of manuscript; Z L had primary responsibility for final content. All authors read and approved the final manuscript.

**Data curation:** Lijuan Wu, Shumei Gong.

**Formal analysis:** Zhenhong Liang, Lijuan Wu.

**Investigation:** Zhenhong Liang.

**Project administration:** Zhenhong Liang, Lijuan Wu.

**Resources:** Shumei Gong, Xiaohong Liu.

**Software:** Xiaohong Liu.

**Writing – original draft:** Zhenhong Liang.

**Writing – review & editing:** Zhenhong Liang.
